# CNX-012-570, a direct AMPK activator provides strong glycemic and lipid control along with significant reduction in body weight; studies from both diet-induced obese mice and db/db mice models

**DOI:** 10.1186/1475-2840-13-27

**Published:** 2014-01-25

**Authors:** Tharappel M Anil, Chandrashekaran Harish, Mudigere N Lakshmi, KrishnaReddy Harsha, Mallappa Onkaramurthy, Venkatesh Sathish Kumar, Nitya Shree, Venkatachalaiah Geetha, Gundalmandikal V Balamurali, Aralakuppe S Gopala, Bobbili Madhusudhan Reddy, Madabosse K Govind, Mammen O Anup, Yoganand Moolemath, Marikunte V Venkataranganna, Madanahalli R Jagannath, Baggavalli P Somesh

**Affiliations:** 1 Connexios Life Sciences Pvt Ltd, Bangalore, India

**Keywords:** AMPK, CNX-012-570, Glucose, Insulin sensitivity, Triglycerides, Cholesterol and body weight

## Abstract

**Objectives:**

AMP activated protein kinase (AMPK) regulates the coordination of anabolic and catabolic processes and is an attractive therapeutic target for T2DM, obesity and metabolic syndrome. We report the anti-hyperglycemic and anti-hyperlipidemic effects of CNX-012-570 is an orally bioavailable small molecule (molecular weight of 530 Daltons) that directly activates AMPK in DIO and *db/db* animal models of diabetes.

**Methods:**

Activity and efficacy of the compound was tested in cell based as well as cell free systems *in vitro*. Male C57BL/6 mice fed with high fat diet (HFD) were assigned to either vehicle or CNX-012-570 (3 mg/kg, orally once a day) for 8 weeks (n = 8). Genetically diabetic *db/db* mice on chow diet were dosed with vehicle control or CNX-012-570 (2.5 mg/kg, orally once a day) for 6 weeks (n = 8).

**Results:**

CNX-012-570 is a highly potent and orally bioavailable compound activating AMPK in both cell and cell free systems. It inhibits lipolysis (33%) and gluconeogenesis (28%) in 3T3L1 cells and rat primary hepatocytes respectively. The efficacy of the molecule was translated to both DIO and *db/db* animal models of diabetes. CNX-012-570 has reduced fasting blood glucose levels by 14%, body weight by 24% and fasting serum triglycerides (TG) by 24%. CNX-012-570 showed a 22% reduction in fed serum cholesterol levels and 19% increase in HDL levels.

In *db/db* mice model, CNX-012-570 has shown 18% decrease in fed glucose and 32% decrease in fasting glucose with a 2.57% reduction in absolute HbA1c. Decrease in serum insulin and glucose AUC indicates the increased insulin sensitivity. Body weight was reduced by 13% with increased browning of adipose tissue and decreased inguinal and mesenteric fat mass. There was significant reduction in liver TG and liver total cholesterol.

**Conclusions:**

CNX-012-570 has the potential to control hyperglycemia and hyperlipidemia. It also reduces body weight gain with an additional benefit of minimizing cardiovascular risks in diabetics.

## Background

AMP- activated Protein Kinase (AMPK) a serine/threonine protein kinase has been postulated as an important drug target in metabolic syndrome and diabetes because of its role in regulation of cellular energy metabolic homeostasis. It is a heterotrimer comprising of a catalytic α, regulatory β and γ subunits [1]. The predominant heterotrimer combination varies from tissue to tissue. The phosphorylation of threonine 172 (Thr 172) at alpha subunit is necessary for activation of AMPK. The initiation of AMPK activation occurs when AMP binds to the γ regulatory subunit followed by conformational changes in α, β and γ [2].

AMPK activation happens in the cell under the low energy conditions (decrease in ATP and increase in AMP). Upon phosphorylation, AMPK drives multiple cellular signaling pathways which can be antagonized by dephosphorylation with protein phosphatases [3-5]. It is known by recent findings that AMPK heterotrimer changes its distribution and localization between cytoplasm and nucleus [6-9] with the help of nuclear export sequence in the catalytic subunit of AMPK [10].

It is well established that AMPK activity is reduced in the diabetic or metabolic syndrome condition [11]. AMPKα2 deletion has shown to reduce insulin sensitivity, impaired glucose tolerance along with defects in insulin secretion [12,13]. Impaired AMPK activation is associated with reduced mitochondrial function and dysregulated intracellular lipid metabolism [14].

In liver, AMPK activation reduces cholesterol synthesis by inhibiting HMGCR [15]. In the adipose tissue, AMPK activation has a direct negative impact on lipolysis through phosphorylation of HSL at Ser 565 and ATGL at Ser 406 [16]. In the skeletal muscle, both leptin [17] and adiponectin [18] activates AMPK whereas exercise mediated AMPK activation depends on local signaling pathways. Apart from its role in metabolism, it is known that AMPK activation is anti-inflammatory and exerts immunosuppressive effects in different cells [19].

In the past, attempts were made to activate AMPK either directly or indirectly in order to get the potential benefit to manage the metabolic syndrome. AICAR (5-aminoimidazole-4-carboxamide ribonucleoside) which mimics AMP thereby activates AMPK whereas metformin activates AMPK by increasing the ratio of AMP/ATP. Many natural compounds like resveratrol and berberine also activate AMPK by changing the nucleotide ratio [20]. Major concern with these indirect activators is that one arm of activation is through AMPK apart from their role in other cellular signaling which is independent of AMPK activation. Small molecule activators like ETC-1002 which apart from inhibiting ATP citrate lyase (ACL-a key enzyme in the cholesterol biosynthetic pathway), activates AMPK which is independent of LKB1 and CAMKKβ [21]. Many attempts were made to activate AMPK directly using specific small molecule activators. One of them is A-769662 [22] which is a β1-subunit specific with no detectable binding or activity towards β2-subunit.

Our understanding of the AMPK target biology revealed that AMPK activation has to be in multiple tissues (like liver, adipose, skeletal muscle, heart, endothelium and pancreas) in order to get the complete benefit of its activation on metabolic syndrome that includes CVD risks. Since each subunit of AMPK exists in different heterotrimer, we need to target all the different heterotrimers in multiple tissues. In this regard, our medicinal chemistry approach was to target both β1 and β2 subunits to activate all the AMPK heterotrimers that are present across multiple tissues. In this study, we report CNX-012-570 (which has an half-life of 5 h in mice) is a dual β1 and β2 subunit specific potent and direct AMPK activator for its potential to control multiple aspects of metabolic syndrome in both diet-induced obese (DIO) and *db/db* mice.

## Methods

### Reagents

3T3L1 (Mouse embryonic fibroblast), C2C12 (Mouse myoblasts cell line) and HepG2 (human liver hepatocytes) cell lines are from ATCC. Antibodies against phospho-AMPKα, AMPKα, phospho-AKT, AKT, phospho-HSL, phospho-mTOR, phospho-JNK, p-eIF2a and β-Actin are from Cell Signaling. Anti-HNF4α antibody is from BioVision. Glucose estimation kit and free glycerol estimation kit from Sigma.

Accu-check glucometer from Roche Diagnostics (Germany) and Ultra-sensitive insulin ELISA kit from Crystal Chem Inc (USA). Triglyceride and Cholesterol estimation reagents are from Diagnostic Systems (Germany). FFA estimation kit from Randox (UK) and Glycerol estimation kits from Sigma. Triton-X and Fluromount were procured from Sigma-Aldrich.

### Cell culture

C2C12 cells were seeded in 24-well plates (3 × 10^4^ cells/well) at 37°C in DMEM containing 25 mM Glucose and 10% FBS. When the cells were confluent, the media was supplemented with 25 mM glucose and 2% FBS for myotubes formation. After 4 days of differentiation and myotubes formation, cells were used for the different treatments.

3 T3-L1 cells were cultured in DMEM supplemented with 10% bovine calf serum (Hyclone, USA) and 25 mM glucose in 96 or 24-well tissue culture plates. To induce differentiation, the culture media was supplemented with 100 nM insulin, 1 μM dexamethasone, and 500 μM isobutylmethylxanthine. Media was changed every 2 days with fresh media containing 100 nM insulin until day 5. The differentiation continued for further 3 days in media without insulin.

HepG2 cells were maintained in MEM media with 10% FBS. Cells were seeded in appropriate culture plates two days prior to the treatment.

### Tissue distribution and AMPK activation studies

Swiss albino mice (10 wk, 30 ± 3 g) were orally dosed with CNX-012-570 (10 mg/kg body wt,) once-daily for 3 days (n = 5). After 3 h of final dose, animals were killed by cervical dislocation. 100 mg of different tissues like liver, adipose and skeletal muscle were collected and homogenized for the analysis of AMPK activation and its downstream target engagement studies using specific antibodies by Western blotting.

### Efficacy studies in disease models

#### ***DIO mice study***

Sixteen week old male C57BL/6 J mice were fed on either high fat diet (HFD) (D12492; 60% kcal from fat; Research Diets, Inc., New Jersey, USA) or chow diet (10% kcal from fat) for 11 weeks. After acclimatization period, animals were selected for the study. Animals were housed in polypropylene cages, maintained at 23 ± 1°C, 60 ± 10% humidity, exposed to 12 h cycles of light and dark and provided ad libitum access to either chow or HFD and water throughout the acclimatization and experimental period. Animal experiment protocols and experimental procedures were approved by the Connexios Institutional Animal Ethics Committee which are in accordance with the ARRIVE guidelines [23].

DIO animals were assigned to specific treatment groups based on body weight, glucose AUC during OGTT, fasting blood glucose and fasting serum TG levels. DIO animals were randomized into control and CNX-012-570 treatment groups (3 mg/kg, orally once a day). Animals (n = 8) fed on normal chow diet were served as lean control. DIO animals in the treatment groups (n = 8) and HFD control group (n = 8) were fed with HFD throughout the experimental period. Animals in the treatment group received CNX-012-570, orally once a day as solution in 10% dimethyl acetamide and 8% cremophor as vehicle for 8 weeks. Lean control and HFD control animals were received vehicle orally once a day. Body weight (weekly) and feed consumption (daily) were recorded. Blood was collected from tail vein for glucose and triglyceride estimation. OGTT was performed on the 8^th^ week of treatment after 6 h fasting with 2 g/kg of oral glucose challenge. After 8 weeks of treatment blood was collected from retro-orbital bleeding for glycerol, free fatty acid, cholesterol, LDL-C estimation. Animals were then euthanized and necropsied; liver was excised immediately, weighed and taken for estimation of triglyceride. Different adipose depots were separated and weighed.

#### ***db/db mice study***

Six week old male *db/db* mice from Jackson Lab were acclimatized for one week and randomized to either vehicle control or CNX-012-570 (2.5 mg/kg, orally once a day) treatment groups (n = 8) based on the body weight, fed glucose and fasting glucose. Age matched db/+ animals served as lean control. Treatment group animals were administered CNX-012-570, orally once a day as solution in 10% dimethyl acetamide and 8% cremophor as vehicle for 6 weeks. Lean control and *db/db* controls animals received vehicle orally once a day. Body weight, fed glucose, fasting glucose (tail snip) were monitored weekly. At the end of the treatment HbA1c (Siemens DCA Vantage system kit) and insulin levels (Downers Grove, USA) were measured. Animals were sacrificed and liver, adipose depots, muscle and serum were collected.

### Fasting and fed glucose

Blood samples were collected by the tail nipping method after 6 h fasting and glucose levels were measured using Accu-check glucometer (Roche diagnostics) once a week during the entire duration of study. Fed state blood samples were collected as mentioned above and glucose levels were measured at every 2 h time intervals upto 16 h and then followed by 4 h intervals upto 24 h.

### Oral glucose tolerance test

OGTT was determined after oral glucose load at a dose of 2 g/kg body weight by oral gavage. Blood glucose was measured by tail clip method at intervals of 0, 15, 30, 60, 90 and 120 min using Accu-check glucometer (Roche diagnostics).

### Fed and fasting insulin

Fed state and 6 h fasted blood samples were collected by the retro-orbital puncture and insulin levels were estimated using Crystal chem kit (ELISA). Homeostasis model assessment of insulin resistance (HOMA-IR) was employed to assess the status of insulin action.

### Serum adiponectin

Blood samples collected at end of the study termination and total high molecular weight adiponectin levels were measured using adiponectin ELISA kit from ALPCO diagnostics according to the manufacturer’s instruction.

### Western blotting

HepG2 cells, C2C12 myotubes and 3T3L1 adipocytes were treated with serum free media containing 0.3 μM of CNX-012-570 and monitored AMPK activation time course for 24 h (0.25, 2,4,6,8,12,16 and 24 h). At each time point, cells were harvested and lysed in lysis buffer. 50 μg of the lysate were separated on 12% SDS-PAGE and transferred to nitrocellulose membrane and probed with primary antibody against phospho-AMPKα and total AMPKα. Signals were developed by enhanced chemiluminescence (West Pico, Thermo Scientific, USA).

For *ex vivo* analysis of protein markers, after the CNX-012-570 treatment period (Swiss albino mice, DIO mice and *db/db* mice), mice were killed by cervical dislocation. 100 mg of tissue from different organs were collected (gastronomes muscle, adipose and liver) in lysis buffer. Lysates were prepared by homogenization and 50 μg of the lysate from each treatment was used for the Western blot analysis for different protein markers as mentioned above.

### Measurement of thermogenesis

Mice were housed individually and transferred to a cold environment with an ambient temperature of 4°C. Rectal temperature was measured for every 15 min for a total of 75 min and animals were then brought to room temperature. The temperature was measured further for 20 min at 10 min intervals.

### Estimation of total cholesterol, LDL-C, glycerol and FFA in serum

Blood was collected from retro orbital under isoflurane anesthesia was allowed to clot for 30 min at room temperature followed by centrifugation for 10 minutes at 4°C and serum was collected for analysis. Serum total cholesterol was measured using fully automated clinical chemistry analyzer EM360, (Transasia Bio-medicals Ltd) with ERBA Kits. LDL-C and glycerol were estimated by colorimetric analysis as per manufacturer’s instruction.

### Estimation of liver TG and cholesterol

Tissue TG was extracted according to Folch’s method. Briefly, lipids were extracted with chloroform: methanol (2:1) mixture, the organic layer was separated and dried in a speed vac. The residue was re-suspended in isopropyl alcohol and assayed for TG and cholesterol levels by using TG and cholesterol quantification kit respectively (Diagnostic systems, Germany).

### Quantitative PCR analysis

After the study termination, 100 mg of liver tissue was collected from each animal across treatment groups in TRIZOL (Sigma, USA). RNA was isolated and later converted to cDNA as per the standard protocol. Relative mRNA levels of SREBP1c (fwd: 5′-AGCAGCCCCTAGAACAAACAC-3′; rev: 5′-CAGCAGTGAGTCTGCCTTGAT-3′), MCP-1 (fwd: 5′- AGCACCAGCCAACTCTCACT-3′; rev: 5′-TCATTGGGATCATCTTGCTG-3′) and APOB100 (fwd: 5′- AAGCACCCCAAGTGTCACAA-3′; rev: 5′- ATTTGTACTGCAGGGCGTCA-3′) were analyzed using SYBR green chemistry.

### Histopathology

Formalin-fixed, paraffin-embedded tissue sections from liver and inguinal adipose depot were sectioned at 4 μm and stained with hematoxylin and eosin (H&E) stain. Histopathological examination was carried out in a blinded fashion using Carl Zeiss Axio Scope A1 microscope. The images were captured at magnification of ×400 using Prog Res C3 camera attached to the microscope.

### Adipose morphometry

Formalin-fixed, paraffin-embedded tissue sections from inguinal adipose depot were sectioned at 4 μm and stained with H&E for adipose morphometry. From each section 10 different randomly selected microscopic fields at ×400 magnification were used for evaluation. Adipose morphometry was carried out using ProgRes Pro, v.2.8.8 image analysis suite. The mean area of adipocytes from all the groups were statistically analyzed using Graphpad Prism, v.5.0.

### Immunofluorescence and image analysis

For UCP1 immunostaining, subcutaneous adipose sections were de-paraffinized and blocked in 1% BSA in PBS for 30 min at RT and incubated with anti-UCP1 (Abcam, USA) primary antibody for 90 min at RT, followed by washing with PBS. Sections were then incubated with secondary antibody (goat anti-rabbit IgG Alexa Fluor 555, Molecular Probes, Invitrogen) for 30 min at RT and mounted using fluromount. Localization of UCP1 was assessed and the images were captured at ×400 magnification. All adipose sections were viewed at ×400 magnification, and images were captured using Zeiss microscope connected via camera to a computer (progres® capture pro 2.1 camera). Adipocytes size measurement was performed using a computer-assisted image analysis progres® capture pro software. From each animal 10 images were captured.

### Statistical analysis

All the values are expressed as mean ± SEM; Students unpaired *t* test was used for comparing cell based assay results. One way analysis of variance was performed followed by Dunnets test for establishing level of significance between treatment and control animals. p < 0.05 was considered as significant.

## Results

### CNX-012-570 activates AMPK in multiple tissues *in vitro* and *in vivo*

**Figure 1:**
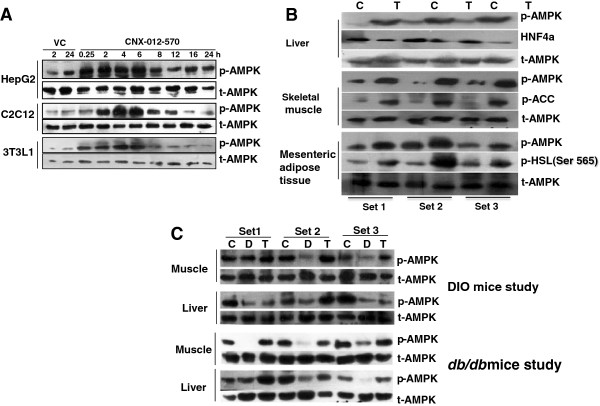
**CNX-012-570 activates AMPK in multiple tissues *in vitro *and *in vivo*: (A) Kinetics of AMPK activation by CNX-012-570 (0.3 μM) as measured by p-AMPK protein levels in HepG2, C2C12 myotubes and 3T3L1 adipocytes. (B)** AMPK activation (p-AMPK protein levels) and its downstream targets proteins phosphorylation (HNF4α, ACC and HSL) from liver, skeletal muscle and adipose tissue respectively from SAM mice after CNX-012-570 treatment (3 doses; 3 mg/kg, orally once a day). **(C)** At the end of the treatment period in both DIO and *db/db* mice, AMPK activation (p-AMPK protein levels) were measured. CNX-012-570 was dosed with 3 mg/kg and 2.5 mg/kg, orally once a day in DIO and *db/db* mice respectively. Blots were developed using specific primary antibodies and HRP conjugated secondary antibody using enhanced ECL reagent as mentioned in the Methods.

CNX-012-570 is a highly potent and direct activator of AMPK with half maximal effective concentration (EC_50_) of 93 nM towards α1β1γ1 isoform (predominant isoform in liver and adipose tissue) and 285 nM towards α2β2γ3 isoform (predominant isoform in skeletal muscle) (Additional file 1A & B) as established using recombinant AMPK heterotrimer complexes. CNX-012-570 activates AMPK in time dependent manner in hepatocytes, muscle cells and in adipocytes as measured by the extent of AMPK phosphorylation (Figure 1A). The activation of AMPK happens as early as 15 min and reaches a maximum at 4-6 h before it comes back to the normal level. In order to test the impact of AMPK activation on cellular outcome, we measured hepatic glucose output in primary rat hepatocytes and isoproterenol mediated lipolysis in 3T3L1 adipocytes. CNX-012-570 inhibits both hepatic glucose output (12-28%) and adipose lipolysis (17-33%) (Additional file 2A & B).

To test AMPK activation and its downstream target engagement in multiple tissues (liver, skeletal muscle and adipose) *in vivo,* Swiss albino mice were treated with CNX-012-570 acutely (3 doses at 10 mg/kg; orally once a day). CNX-012-570 activates AMPK in all the tested tissues and shown its downstream target engagement as measured with HNF4a phosphorylation degradation in liver, phosphorylation of ACC in skeletal muscle and HSL in adipose (Figure 1B). We have also tested the AMPK activation in both DIO mice on HFD and *db/db* mice. AMPK activation in all the tissues (liver, skeletal muscle and adipose) is reduced in disease model compared to control animals. Treatment with CNX-012-570 restored the AMPK activation in both DIO and *db/db* mice disease models (Figure 1C).

### CNX-012-570 improves insulin sensitivity and glucose tolerance

**Figure 2:**
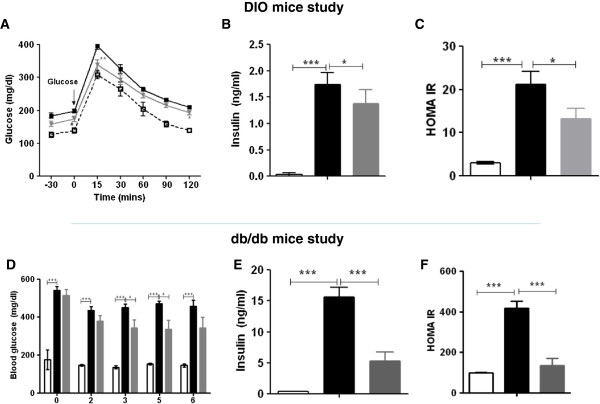
**CNX-012-570 improves insulin sensitivity and glucose tolerance in DIO and *db/db *mice. (A)** OGTT in DIO mice on week 8 of treatment. Open square-Lean control, closed square -HFD control and Triangle-CNX-012-570 (3 mpk, qd). **(D)** Fed glucose levels in *db/db* mice study were monitored weekly. **(B & E)** Fasting insulin, **(C & F)** HOMA IR in both DIO and *db/db* mice were measured at week 8 and 6 respectively. Clear bar-lean control, black bar-HFD or *db/db* control, grey bar-CNX-012-570 treatment (3 and 2.5 mg/kg, orally once a day in DIO and *db/db* mouse respectively). All the values are expressed as Mean ± SEM. Statistical comparison between control and treatment group was conducted by One-way ANOVA followed by Dunnett’s test (*P < 0.05, ** P < 0.01 and *** P < 0.001).

We tested impact of AMPK activation by CNX-012-570 on glucose intolerance in HFD mice. The data indicates an improvement in glucose intolerance as measured by an oral glucose tolerance test. Upon chronic CNX-012-570 treatment, we observed ~10% decrease in glucose excursion as compared to HFD mice (glucose AUC of 29736 ± 775 in CNX-012-570 treated Vs 32718 ± 680 in HFD control; P < 0.05) (Figure 2A). We observed a significant increase in fasting insulin along with HOMA-IR in HFD animals compared to lean control (1.74 ± 0.23 vs 0.03 ± 0.04 ng/ml; P < 0.001 for insulin and 27.17 ± 3.04 vs 2.99 ± 0.34; P < 0.001 for HOMA-IR respectively). Treatment with CNX-012-570 reduced both fasting insulin and HOMA-IR by ~21% and 38% respectively as compared to HFD control (1.38 ± 0.26 vs 1.74 ± 0.23 ng/ml; P < 0.05 for insulin and 13.17 ± 0.34 vs 27.17 ± 3.04; P < 0.01 for HOMA-IR) (Figure 2B & C).

In *db/db* control mice, fed glucose levels were significantly high (~450 ± 20 vs ~145 ± 6 mg/dl; P < 0.001) as compared to lean control animals. Treatment with CNX-012-570 has reduced fed glucose non-significantly (~15%) from week 2 and reached a significant reduction of ~25% after week 3 as compared to *db/db* control animals (343 ± 42 vs 450 ± 20 mg/dl; P < 0.05). This reduction was maintained during the entire treatment period (Figure 2D). *db/db* control animals have shown a high levels of insulin (15.6 ± 1.6 vs 0.4 ± 0.0 ng/ml; P < 0.001) and HOMA-IR (417.73 ± 35.43 vs 3.6 ± 0.44; P < 0.001) compared to control animals. CNX-012-570 treated *db/db* animals decreased fasting insulin by ~66% (5.3 ± 1.4 vs 15.6 ± 1.6 ng/ml; P < 0.001) and HOMA-IR by 68% as compared *db/db* animals (135.43 ± 35.18 vs 417.73 ± 35.43; P < 0.001) (Figure 2E & F).

### CNX-012-570 reduces blood glucose, HbA1c, glycerol and FFA

**Figure 3:**
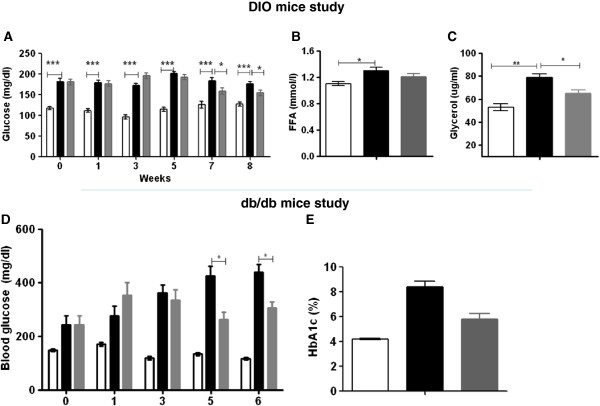
**CNX-012-570 reduces blood glucose, glycerol and FFA: (A & D) Fasting Glucose levels were monitored weekly in both DIO and *db/db* mice respectively. At the end of treatment period, Fasting Serum FFA (B) and glycerol levels (C) were measured in DIO mice. (E)** HbA1c levels in *db/db* mice at week 6. Clear bar-lean control, black bar-HFD or *db/db* control, grey bar-CNX-012-570 treatment (3 and 2.5 mg/kg, orally once a day in DIO and *db/db* mouse respectively). All the values are expressed as Mean ± SEM. Statistical comparison between control and treatment group was conducted by One-way ANOVA followed by Dunnett’s test (*P < 0.05, ** P < 0.01 and *** P < 0.001).

HFD control animals exhibited a significantly high levels of fasting glucose as compared to lean control from week 1 and maintained during the entire study period (~180 ± 6 vs ~114 ± 5 mg/dl; P < 0.001). Treatment with CNX-012-570 started reducing the fasting glucose from week 7 and maintained the same on week 8. The reduction is 15% as compared to HFD control (155 ± 7 vs 180 ± 6 mg/dl; P < 0.05) (Figure 3A). Also we observed a significant 15% decrease in serum glycerol (65 ± 3 vs 79 ± 3 μg/ml; P < 0.05) and free fatty acid (1.09 ± 0.07 vs 1.30 ± 0.05 mmol/l; P < 0.05) with CNX-012-570 treatment as compared to HFD control (Figure 3B & C).

In *db/db* mice, fasting glucose was very high from week 1 and reachesd a maximum at week 6 compared to control animals (439 ± 30.9 vs ~130 ± 7 mg/dl; P < 0.001). Treatment with CNX-012-570 reduced fasting glucose significantly by 32% (439 ± 30.9 vs 306 ± 22.9 mg/dl; P < 0.01) compared to *db/db* control and the decrease was observed from week 3 (Figure 3D). At the end of the study, HbA1c levels were reduced significantly by an overall 2.6% with CNX-012-570 treatment (32% of HFD control animals; i.e. 5.83 ± 0.44 vs 8.34 ± 0.42; P < 0.001) (Figure 3E).

### CNX-012-570 reduces serum triglyceride, total cholesterol and has a potential to reduce macro-vesicular steatosis

**Figure 4:**
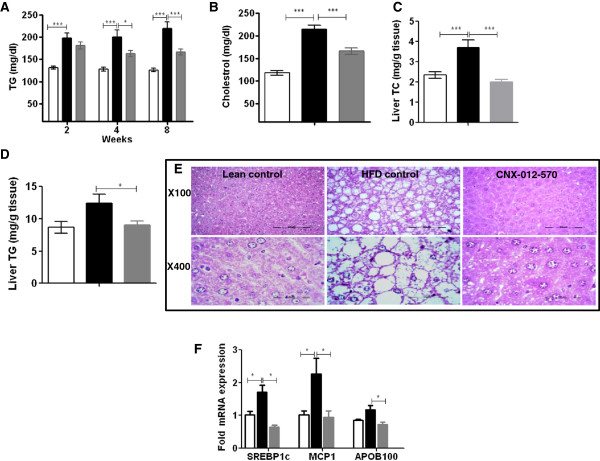
**CNX-012-570 reduces serum triglyceride, total cholesterol and has a potential to reduce macro-vesicular steatosis in DIO mice. (A)** Fasting serum triglycerides were measured once in two weeks. **(B)** At week 8, serum fed cholesterol was measured. **(C & D)** After the study termination, liver cholesterol and triglyceride levels were measured. **(E)** Histopathological analysis of formalin fixed liver sections after H&E staining. **(F)** After the study termination, mRNA levels of liver SREBP1c, MCP1 and APOB100 were measured as mentioned in materials and methods. Clear bar-lean control, black bar-HFD control, grey bar-CNX-012-570 treatment (3 mg/kg, orally once a day). All the values are expressed as Mean ± SEM. Statistical comparison between control and treatment group was conducted by One-way ANOVA followed by Dunnett’s test or students unpaired t-test as appropriate (*P < 0.05, ** P < 0.01 and *** P < 0.001).

Compared to lean control animals, HFD animals have shown a significant increase in serum triglyceride levels (120 ± 4 vs 220 ± 14 mg/dl; P < 0.01). This trend was maintained during the entire study period. Treatment with CNX-012-570 reduced serum triglycerides from week 2 and reached a maximum of 24% on week 8 compared to HFD control (166 ± 7 vs 220 ± 14 mg/dl; P < 0.001) (Figure 4A). Also we observed a significant increase in serum cholesterol levels in HFD control animals than that of lean control (214 ± 9 vs 118 ± 5 mg/dl; P < 0.001). CNX-012-570 treated animals have shown a 22% significant decrease in serum cholesterol than that of HFD control (116 ± 7 vs 214 ± 9 mg/dl; P < 0.001) (Figure 4B). In this study we observed a non-significant 8% reduction LDL levels (data not shown). After the study termination, we measured liver triglycerides and cholesterol. Triglyceride levels were high in the HFD control than lean control (12.4 ± 1.4 vs 8.6 ± 0.9 mg/g tissue; P < 0.05). CNX-012-570 reduced liver triglycerides significantly by 27% as compared to HFD control (9 ± 0.6 vs 12.4 ± 1.4 mg/g tissue; P < 0.01) (Figure 4D). Liver cholesterol levels were reduced by 40% in CNX-012-570 treated animals than that of HFD control (2 ± 0.12 vs 3.7 ± 0.4 mg/g tissue; P < 0.01) (Figure 4C).

After the study termination, histological evaluation of liver from all the 3 groups was performed. HFD control animals have shown a significant increase in macro-vesicular steatosis with larger lipid droplets as compared to lean control. In CNX-012-570 treated animals, a significant reduction in macro-vesicular steatosis was observed (Figure 4E). mRNA levels of liver steatosis markers like SREBP1c (stimulate liver lipid synthesis), monocytes chemoattractant protein-1 (MCP1, a proinflammatory cytokine) and APOB100 (apolipoprotein of chylomicrons and low-density lipoproteins (LDL)) were reduced in CNX-012-570 treatment animals as compared to HFD control animals (Figure 4F).

### CNX-012-570 reduces body weight

**Figure 5:**
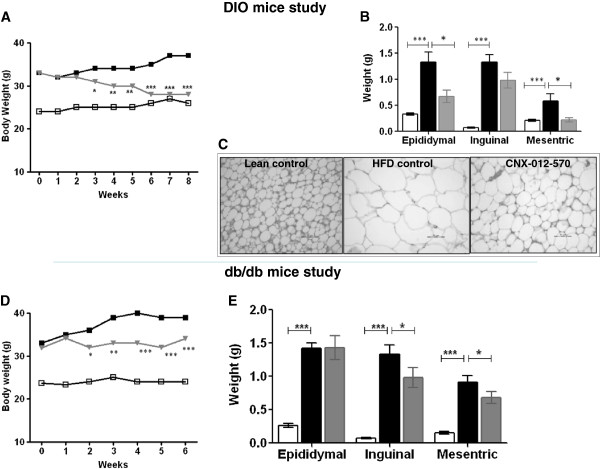
**CNX-012-570 reduces body weight: (A & D) Body weight from both the treatment group was measured weekly.** Open square-Lean control, closed square-HFD or *db/db* control and Triangle-CNX-012-570 treatment. **(B & E)** At end of the treatment period, different adipose depot weight was measured from both DIO and *db/db* mice study respectively. **(C)** Histology of inguinal adipose tissue sections were analyzed after H&E staining. Clear bar-lean control, black bar-HFD or *db/db* control, grey bar-CNX-012-570 treatment (3 and 2.5 mg/kg, orally once a day in DIO and *db/db* mouse respectively). All the values are expressed as Mean ± SEM. Statistical comparison between control and treatment group was conducted by One-way ANOVA followed by Dunnett’s test (*P < 0.05, ** P < 0.01 and *** P < 0.001).

The change in body weight in the lean control during the study period was not significant except a small increase after week 6 (24.6 g to 26.6 g). HFD animals have shown a steady increase in body during the study period and attain a maximum after week 7 (33 g to 37 g). CNX-012-570 treated animals started showing a significant decrease in body weight from week 3 (11%) and reached a maximum of 24% on week 6 and maintained the same till week 8 as compared to HFD animals (Figure 5A). During the course of the study, feed consumption was recorded weekly and did not observe any change in the consumption rate across the treatment groups (data not shown). Also we observed a significant 40-50% decrease in adipose depot weight in CNX-012-570 treated animals than that of HFD animals (Figure 5B). Morphometry analysis of inguinal adipose depot revealed a significant decrease in adipocytes size in CNX-012-570 treated animals as compared to the HFD control animals (Figure 5C).

In *db/db* animals, the body weight gain was significant from week 1 to week 6 (33 g to 39 g) whereas control animals maintained a steady body weight during the study period (23.6 g on week 1 to 24 g on week 6). Treatment with CNX-012-570 started decreasing body weight significantly on week 2 (10%) and reached a maximum decrease of 16% on week 5 and maintained till week 6 (Figure 5D) without change in the feed consumption during the study period (data not shown). At the end of the study, we measured the adipose depot weight and found CNX-012-570 treatment decreased 20% of both mesenteric and inguinal adipose depot weight (Figure 5E).

### CNX-012-570 improves insulin signaling and reduces stress

**Figure 6:**
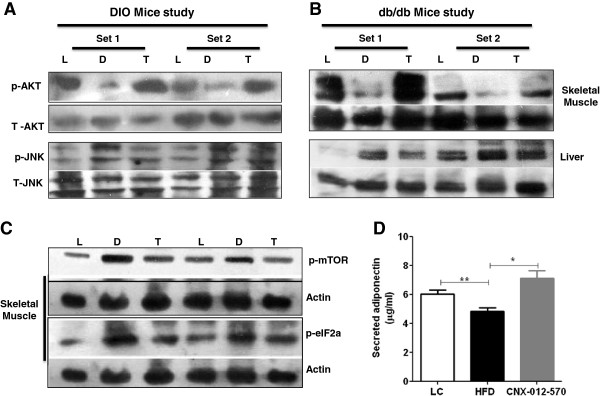
**CNX-012-570 improves insulin signaling and reduces stress in DIO and *db/db *mice models: At the end of the treatment period in both DIO and *db/db* mice, both liver and skeletal muscle tissues were collected for the analysis of both insulin sensitivity and stress markers. (A & B)** phospho-AKT levels in skeletal muscle (upper panel) and phospho-JNK levels in liver (lower panel) from both DIO and *db/db* mice study respectively. **(C)** phospho-mTOR (upper panel) and phospho-eIF2α (lower panel) levels from skeletal muscle from DIO mice. L- Lean control, D- DIO or *db/db* control, T- CNX-012-570 treatment. **(D)** Serum adiponectin levels from *db/db* mice study. Clear bar-lean control, black bar-*db/db* control, grey bar-CNX-012-570 treatment (2.5 mg/kg, orally once a day). Statistical comparison between control and treatment group was conducted by One-way ANOVA followed by Dunnett’s test (*P < 0.05, ** P < 0.01 and *** P < 0.001).

Insulin sensitivity was assessed by measuring phospho-AKT in both DIO mice and *db/db* mice. When compared to lean control, both HFD control and *db/db* control animals showed a decrease in phospho-AKT by 40-50%. Treatment with CNX-012-570 restored the phospho-AKT levels to lean control level and the insulin sensitivity (top panel, Figure 6A&B). Also we observed a reduction in serum adiponectin levels which was restored to lean control level by the CNX-012-570 treatment (Figure 6D). Phospho-JNK in liver in both the disease models, observed a significant 2-3 fold increase in phospho-JNK levels as compared to respective lean control. Treatment with CNX-012-570 reduced the phospho-JNK levels which are comparable to the respective lean control (bottom panel, Figure 6A&B). Also we have observed a significant ~2 fold increase in both phospho-eIF2α and phospho-mTOR levels in the HFD control animals. Treatment with CNX-012-570 reduced both phospho-eIF2α and phospho-mTOR levels significantly (Figure 6C).

### CNX-012-570 enhances non-shivering thermogenesis

**Figure 7:**
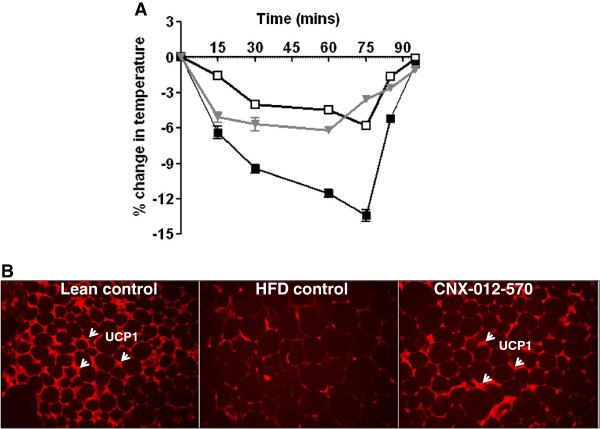
**CNX-012-570 enhances non-shivering thermogenesis (DIO): (A) Non-shivering thermogenesis was measured as described in the Methods.** Open square-Lean control, closed square-HFD control and triangle-CNX-012-570 treatment. **(B)** Immunohistochemistry of UCP1 in subcutaneous adipose tissue sections as mentioned in the Methods.

We observed a similar basal body temperature in all the animals from 3 groups. After moving the animals to 4°C, the drop in the temperature was ~5-6% in both HFD control and CNX-012-570 treated animals after 15^th^ min as compared to lean control. After 30^th^min, there was further drop in temperature in HFD animals and continued till 75^th^min (the drop was ~14%) before it came back to normal after 90^th^min. On the other hand, CNX-012-570 treated animals maintained a drop of 5% till 60^th^min and were equivalent to lean control after 70^th^min (Figure 7A). After observing an adaptive mechanism for the cold temperature by CNX-012-570 treated animals, we measured the expression of UCP1 in the inguinal fat. There was a significant decrease in the UCP1 expression in HFD control animals whereas CNX-012-570 treated animals restored the expression. The increase in the UCP1 expression was corroborated with decrease in adipocytes size in CNX-012-570 treated animals as compared to HFD control (Figure 7B).

## Discussion

In the present study, we have used a direct and selective AMPK activator CNX-012-570 to evaluate its potential to modulate the various metabolic abnormalities in both DIO mice on HFD and *db/db* mice. These metabolic abnormalities were monitored weekly over the period of 6 and 8 weeks in DIO and *db/db* mice respectively.

AMPK activation has been reported by various mechanisms. AICAR activates AMPK after converting to ZMP which mimics the effect of AMP [24]. A-769662 and salicylate activate AMPK by binding to glycogen binding domain on β1-subunit [25,26]. Since CNX-012-570 is not an AMP mimetic and did not change nucleotide levels like metformin [27] (Additional file 3A & B), this activation is a direct interaction of the compound with the AMPK heterotrimeric complex. Since CNX-012-570 is a dual β1 and β2 agonist, we expect the compound activates all AMPK heterotrimers across the tissues. This has been substantiated by our data both *in vitro* and *ex vivo* from multiple cell types (hepatocytes, muscle cells, adipocytes, endothelial cells and macrophages) and tissues (liver, skeletal muscle and adipose tissue) respectively. Also our data clearly shows that AMPK activation triggered the downstream signaling (in terms of its downstream target engagement: phosphorylation of ACC and HSL) which is necessary to bring changes in the metabolic state of each tissue.

### Impact on hyperglycemia and insulin sensitivity

In both the animal models of disease, it is evident that AMPK activation in liver and adipose is significantly compromised. Chronic activation of AMPK (6-8 weeks) has shown a significant decrease in both fasting plasma glucose and insulin in both animal models of insulin resistance. Also, decreased gluconeogenic substrates like FFA and glycerol from adipose (also seen inhibition of lipolysis in vitro; supplementary data) and alanine (data not shown) from muscle support the decrease in fasting glucose levels. One of the known mechanisms of controlling fasting glucose by AMPK is by repressing the HNF4α mediated transcription of gluconeogenic genes in liver [15]. Apart from decreasing hepatic gluconeogenesis in rat primary hepatocytes (supplementary data), we also observed a significant decrease in total HNF4α levels by CNX-012-570 (data not shown) indicating its direct role in controlling fasting glucose.

Improved insulin sensitivity (reduced hyperinsulinemia) with CNX-012-570 was translated into reduced glucose intolerance and fed glucose levels in both DIO mice and *db/db* mice study respectively. We observed reduction in fed glucose levels from week 2 of the treatment in *db/db* mice. We observed a good 24 h glucose control (feed was provided all the time, data not shown) upon CNX-012-570 treatment in *db/db* study confers the overall improvement in glucose tolerance. This kind of acute control was observed with AICAR. Though the effect is partly due to AMPK activation, most of the control was attributed to its ability to inhibit fructose-1, 6-bisphosphatase (a key enzyme in gluconeogenic pathway) by ZMP [28]. Also we observed a significant increase in the phospho-AKT levels in both the animal models upon AMPK activation indicating improved insulin sensitivity/signaling in skeletal muscle and thus support the mechanistic root for the control of fed glucose. A similar kind of improved insulin sensitivity was observed when activation of AMPK/SIRT1 signaling pathway by telmisartan in skeletal muscle [29]. Hyperinsulinemia is known to increase mTOR/p70 S6K pathway and increased IRS-1/2 serine phosphorylation indicating insulin resistance state of a tissue [30]. Also increase phopsho-eIF2α levels are associated with increased glucose intolerance and insulin resistance [31]. Treatment with CNX-012-570 reduced hyper-phosphorylated mTOR and phopsho-eIF2α indicating an improvement in insulin sensitivity and normalization of protein synthesis. We observed a significant reduction in liver phospho-JNK indicating improved liver insulin sensitivity also. Serum adiponectin levels were reduced in *db/db* mice and observed a significant improvement upon CNX-012-570 treatment. This was reflected in significant reduction in HOMA-IR and HbA1c levels. These data from different tissues indicates that CNX-012-570 improves whole body insulin sensitivity through multiple mechanisms.

### Impact on dyslipidemia and nonalcoholic fatty liver disease (NAFLD)

In DIO mice, treatment with CNX-012-570 has reduced circulating TG levels significantly along with a modest reduction serum FFA. The reduction in TG was appreciated at week 2 of treatment. Also this could be due to decrease in adipose lipolysis, decreased storage or increased fat oxidation in liver. Our data clearly demonstrates that CNX-012-570 inhibits adipose lipolysis and *ex vivo* studies from liver clearly shows a reduction in expression of SREBP1c mRNA levels and other lipogenic markers indicating decreased lipogenesis. The role of AMPK in regulating lipogenesis by reducing both activity and expression of SREBP1c is well established [32,33]. Treatment with CNX-012-570 decreased serum fed cholesterol and the liver cholesterol significantly in the DIO mice on HFD. The decrease in liver cholesterol is a direct consequence of AMPK activation by CNX-012-570. It is well known that AMPK activation leads to decrease in cholesterol biosynthesis. It happens at two levels, one at the transcriptional level where AMPK reduces SREBP1c transcription [32] which is known to transcribe HMG-CoA reductase (HMGCR) a rate-limiting enzyme in cholesterol synthesis. The other regulation is at the inactivation of HMGCR activity by phosphorylation [33,34]. Our data clearly demonstrates that CNX-012-570 decreases SREBP1c and APOB100 mRNA levels in liver and increases phospho-HMGCR levels in HepG2 cells (supplementary data). Even though further studies in appropriate animal models are necessary, these data indicates that CNX-012-570 potential to reduce hypercholesterolemia.

NAFLD starts with fatty liver (hepatic steatosis), can progress to nonalcoholic steatohepatitis (NASH) and end up with liver fibrosis and cirrhosis. In metabolic syndrome that includes obesity, type 2 diabetes and dyslipidemia (elevated TG levels) are associated with NAFLD/NASH [35]. It is proven in humans that treatment of NAFLD with a good insulin sensitizer like metformin has a favorable benefit towards hepatic steatosis. It is speculated that the effect seen may be independent of its glycemic controlling mechanisms [36]. Even though metformin effect on glycemic control is known to be by AMPK dependent/independent activation, the mechanism of metformin action in NAFLD is not established. In HFD control mice, we observed a significant increase in both liver TG and serum TG. The increase in liver TG is associated with increase in macro vesicular steatosis in the HFD control animals. Treatment with CNX-012-570 decreased both liver TG and macro vesicular steatosis significantly. Increased expression of both lipogenic marker SREBP1c and proinflammatory cytokine MCP-1 are known to induce liver steatosis [37,38]. AMPK activation with CNX-012-570 reduced both SREBP1c and MCP-1 mRNA levels significantly. These results indicate a direct role of AMPK in controlling the hepatic steatosis. Some more studies are needed to strengthen AMPK role in NASH and liver fibrosis.

### Impact on body weight and energy expenditure

In our both DIO and *db/db* mice study, the weight of disease control animals increased progressively during the study period. Treatment with CNX-012-570 reduced the body weight gain in both the models significantly. In fact we observed a 10% decrease in body weight in DIO mice study. The body weight reduction was observed at week 2 of treatment. This kind reduction has observed only when there is an enhanced fat oxidation by physical activity, marked improvement in insulin sensitivity, normal adiponectin levels and decreased depots of adipose tissue [39,40]. When we measured the weight of each depot, there was a significant decrease in each depot weight in CNX-012-570 treated animals except in epididymal depot of *db/db* treated animal. Apart from decreased adipose depots, there was a significant reduction in inguinal adipocytes size. AMPK activation by AICAR is known to increase uncoupling protein-1 (UCP1) expression in white adipose tissue and increase thermogenesis, which can potentially increase energy expenditure and fat oxidation [41]. In DIO mice study, apart from slow recovery the drop in the rectal temperature was significant in the HFD control animals compared to the lean control. CNX-012-570 treated animals recovered very quickly like the lean control animals and the drop was not significant as compared to HFD animals indicating enhanced energy expenditure and fat oxidation. Increased UCP1 protein staining substantiated our claim further that CNX-012-570 treatment has a potential to increase the browning phenotype of white adipose tissue.

### Future direction

Recent studies have shown that glycocalyx layer has been damaged or degraded in chronic hyperglycemia [42]. Metformin treatment has shown a clear improvement in the glycocalyx barrier [43]. Since most of the metformin effects are through AMPK activation, impact of CNX-012-570 on improvement in glycocalyx barrier will be assessed in the later studies.

In heart, AMPK activation is known to protect cardiomyocytes apoptosis by regulating cardiac autophagy [44]. AMPK activation also protects heart during myocardial ischemia-reperfusion injury by activating the pro-survival kinases like AKT, ERK1/2 and GSK3ß [45]. Our preliminary results have shown that CNX-012-570 protects heart from myocardial ischemia-reperfusion injury (manuscript in preparation).

## Conclusions

In summary, CNX-012-570 is a direct and selective, orally bioavailable dual β1 & β2 AMPK activator. Pharmacological activation of AMPK with CNX-012-570 can give strong glycemic control and improve insulin sensitivity. It can reduce serum and tissue lipid levels significantly with a potential control the hepatic steatosis. Some more independent studies need to be performed to evaluate CNX-012-570 potential as a stand-alone anti-dyslipidemic agent. CNX-012-570 has a potential to reduce body weight.

CNX-012-570 is an early lead in our discovery program. CNX-012-570 is a safe, metabolically stable with no risk of drug-drug interaction. Long-term safety and toxicity studies in multiple species will be necessary to progress for studies in humans.

## Abbreviations

AMPK: AMP- 5′ AMP-activated protein kinase; OGTT: Oral glucose tolerance test; AICAR: 5-amino-1-β-D-ribofuranosyl-imidazole-4-carboxamide; HFD: High fat diet; T2DM: Type 2 diabetes mellitus; DIO: Diet induced obesity; UCP1: Uncoupling protein 1; HMGCR: 3-hydroxy-3-methyl-glutaryl-CoA reductase; SREBP1c: Sterol regulatory element-binding protein 1c; TG: Triglyceride; LDL: Low-density lipoprotein; ACC: Acetyl-CoA carboxylase; ACL: ATP citrate lyase; ATP: Adenosine triphosphate; CAMKKB: Calcium/calmodulin-dependent protein kinase kinase beta; FFA: Free fatty acid; LKB1: Liver kinase beta1; MCP1: Monocyte chemoattractant protein-1; NAFLD: Non-alcoholic fatty liver disease; NASH: Nonalcoholic steatohepatitis; PBS: Phosphate buffered saline.

## Competing interests

All the authors are employees of Connexios Life Sciences Pvt Ltd and declare that they have no competing interests.

## Authors’ contributions

CH, MNL, KH, MO, VS, NS, VG, GVB, ASG, RM, carried out experiments; TMA, MKG, AMO, YM, MVV, SBP and JMR planned/executed the study and analyzed data. SBP wrote the manuscript. All authors read and approved the final manuscript.

## Supplementary Material

Additional file 1 CNX-012-570 activates both β1 and β2 sub-unit containing AMPK heterotrimer. Click here for file

Additional file 2 CNX-012-570 mediated activation of AMPK inhibits both hepatic glucose output and adipose lipolysis. Click here for file

Additional file 3 CNX-012-570 is a direct activator of AMPK and not an AMP mimetic. Click here for file
