# Supplementary material for: CNX-012-570, a direct AMPK activator provides strong glycemic and lipid control along with significant reduction in body weight; studies from both diet-induced obese mice and db/db mice models
Source: Cardiovasc Diabetol. 2014 Jan 25;13:27. doi: 10.1186/1475-2840-13-27 (PMC3906767; doi:10.1186/1475-2840-13-27)
Supplement: Additional file 1 — CNX-012-570 activates both β1 and β2 sub-unit containing AMPK heterotrimer. [file 1475-2840-13-27-S1.doc]

**Additional file 1**

**A**

**B**

**Sup. Fig S1: CNX-012-570 activates both β1 and β2 containing AMPK heterotrimer.** The assay was done at Signal Chem, Canada using SAMS peptide and radio labeled ATP in recombinant A1B1G1 and A2B2G3 isoform. EC_50_ was calculated using graph pad prism. **A**: Activity of CNX-012-570 with A1B1G1 isoform of AMPK. **B**: Activity of CNX-012-570 with A2B2G3 isoform of AMPK.
