# Supplementary material for: CNX-012-570, a direct AMPK activator provides strong glycemic and lipid control along with significant reduction in body weight; studies from both diet-induced obese mice and db/db mice models
Source: Cardiovasc Diabetol. 2014 Jan 25;13:27. doi: 10.1186/1475-2840-13-27 (PMC3906767; doi:10.1186/1475-2840-13-27)
Supplement: Additional file 2 — CNX-012-570 mediated activation of AMPK inhibits both hepatic glucose output and adipose lipolysis. [file 1475-2840-13-27-S2.doc]

**Additional file-3**

**
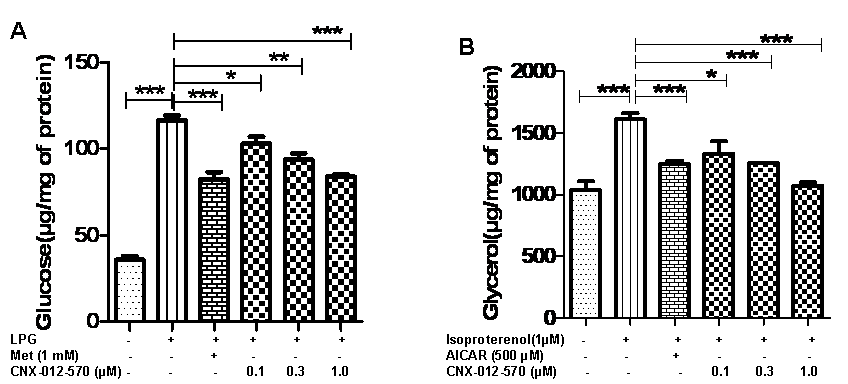
**

**Sup Fig S3: CNX-012-570 mediated activation of AMPK inhibits both hepatic glucose output and adipose lipolysis.**

**A**: **CNX-012-570 inhibits hepatic glucose output.** Rat primary hepatocytes were isolated according to the standard protocol and were treated gluconeogenesis inducing media containing 5mM lactate (L), 5mM pyruvate (P) and 0.5% glycerol (G) in glucose free phenol red free DMEM (PRFM) and incubated for 18h in the absence and presence of indicated concentrations of CNX-012-570. Glucose released in the media was measured using GOD method (Diagnostic Systems) and normalized with total protein. Metformin (Met) was kept as positive control. **B**: **CNX-012-570 inhibits lipolysis in adipocytes**. Fully differentiated 3T3L1 cells were induced with isoproterenol in PRFM in the absence and presence of indicated concentrations of CNX-012-570 for 18 h. Glycerol released in the medium was measured using Free Glycerol Reagent (Sigma, USA) and normalized with total protein. AICAR was kept as positive control. Statistical analysis was done using unpaired students *t* test. **p*<0.05, ** *p*<0.01, ****p*<0.001
