# Supplementary material for: CNX-012-570, a direct AMPK activator provides strong glycemic and lipid control along with significant reduction in body weight; studies from both diet-induced obese mice and db/db mice models
Source: Cardiovasc Diabetol. 2014 Jan 25;13:27. doi: 10.1186/1475-2840-13-27 (PMC3906767; doi:10.1186/1475-2840-13-27)
Supplement: Additional file 3 — CNX-012-570 is a direct activator of AMPK and not an AMP mimetic. [file 1475-2840-13-27-S3.doc]

**Additional file 2**

**A**

**B**

**Sup. Fig S2: CNX-012-570 is a direct activator of AMPK and not an AMP mimetic.**

**A**: **Effect of CNX-012-570 on glycogen phosphorylase activity in a cell free system**. Enzymatic reaction contains phosphate buffer (pH 6.8), MgSO4, β-NADP, Glucose-6-Phosphate dehydrogenase, Phosphoglucomutase and Glycogen phosphorylase-β (Sigma) with or without AMP or with 1μM of CNX-012-570. The reaction was initiated by adding glycogen as a substrate and NADPH produced is measured at 340 nm at different time points as indicated in the graph. AMP was used as positive control. **B**: **Effect of CNX-012-570 on AMP: ATP ratio.** Rat primary hepatocytes were treated with 1 uM of CNX-012-570 for 4 h in serum free DMEM media. Nucleotides were extracted using 0.3 M perchloric acid and neutralized with KOH. Nucleotides were quantified with LCMS/MS. For preparation of standard curve, endogenous nucleotides were denatured by treating with Acid phosphatase. AICAR (100 µM) was used as positive control. Statistical analysis was done using unpaired students *t* test. **p*<0.05, ** *p*<0.01, ****p*<0.001.
